# Single-Cell RNA Sequencing of Retina:New Looks for Gene Marker and Old Diseases

**DOI:** 10.3389/fmolb.2021.699906

**Published:** 2021-07-30

**Authors:** Peixi Ying, Chang Huang, Yan Wang, Xi Guo, Yuchen Cao, Yuxi Zhang, Sheng Fu, Lin Chen, Guoguo Yi, Min Fu

**Affiliations:** ^1^ The Second Clinical School, Southern Medical University, Guangzhou, China; ^2^ Eye Institute and Department of Ophthalmology, Eye & ENT Hospital, Fudan University, Shanghai, China; ^3^ NHC Key Laboratory of Myopia, Fudan University, Shanghai, China; ^4^ Key Laboratory of Myopia, Chinese Academy of Medical Sciences, Shanghai, China; ^5^ Shanghai Key Laboratory of Visual Impairment and Restoration, Shanghai, China; ^6^ Department of Ophthalmology, South China Hospital, Health Science Center, Shenzhen University, Shenzhen, China; ^7^ Medical College of Rehabiliation, Southern Medical University, Guangzhou, China; ^8^ The University of South China, Hengyang, China; ^9^ Department of Anesthesiology, Shenzhen Hospital, Southern Medical University, Shenzhen, China; ^10^ Department of Ophthalmology, the Sixth Affiliated Hospital of Sun Yat-sen University, Guangzhou, China; ^11^ Department of Ophthalmology, Zhujiang Hospital, Southern Medical University, Guangzhou, China

**Keywords:** single-cell RNA sequencing, ScRNA-seq, retina, gene, retinal disease

## Abstract

The retina is composed of 11 types of cells, including neurons, glial cells and vascular bed cells. It contains five types of neurons, each with specific physiological, morphological, and molecular definitions. Currently, single-cell RNA sequencing (sRNA-seq) is emerging as one of the most powerful tools to reveal the complexity of the retina. The continuous discovery of retina-related gene targets plays an important role in helping us understand the nature of diseases. The revelation of new cell subpopulations can focus the occurrence and development of diseases on specific biological activities of specific cells. In addition, sRNA-seq performs high-throughput sequencing analysis of epigenetics, transcriptome and genome at the single-cell level, with the advantages of high-throughput and high-resolution. In this paper, we systematically review the development history of sRNA-seq technology, and summarize the new subtypes of retinal cells and some specific gene markers discovered by this technology. The progress in the diagnosis of retinal related diseases is also discussed.

## Introduction

With the development of high-throughput sequencing technology, humans can already analyze genomes and their products on a large scale, including DNA sequences, chromatin structure, RNA transcripts, proteins and metabolites (Botond, 2018). Traditional high-throughput sequencing requires sufficient DNA samples to be obtained from a large number of cells. However, the accuracy of high-throughput sequencing is quite low, and the result of sequencing should be corrected. Single-cell RNA sequencing (scRNA-seq) refers to the technology of high-throughput sequencing analysis of the genome, transcriptome and epigenetic genome at the single cell level. Currently, scRNA-seq technology is commonly used in the fields such as development of stem cell, embryo and tumor. For example, in the study of tumor tissues, researchers classify subgroups based on single-cell transcription maps (Masland, 2012; Wang et al., 2014), and based on the gene expression profiles, they can study the mechanism of cancer cell metastasis (Zheng et al., 2017) and discover new targets for immunotherapy (Pauly et al., 2019).

**FIGURE 1:**
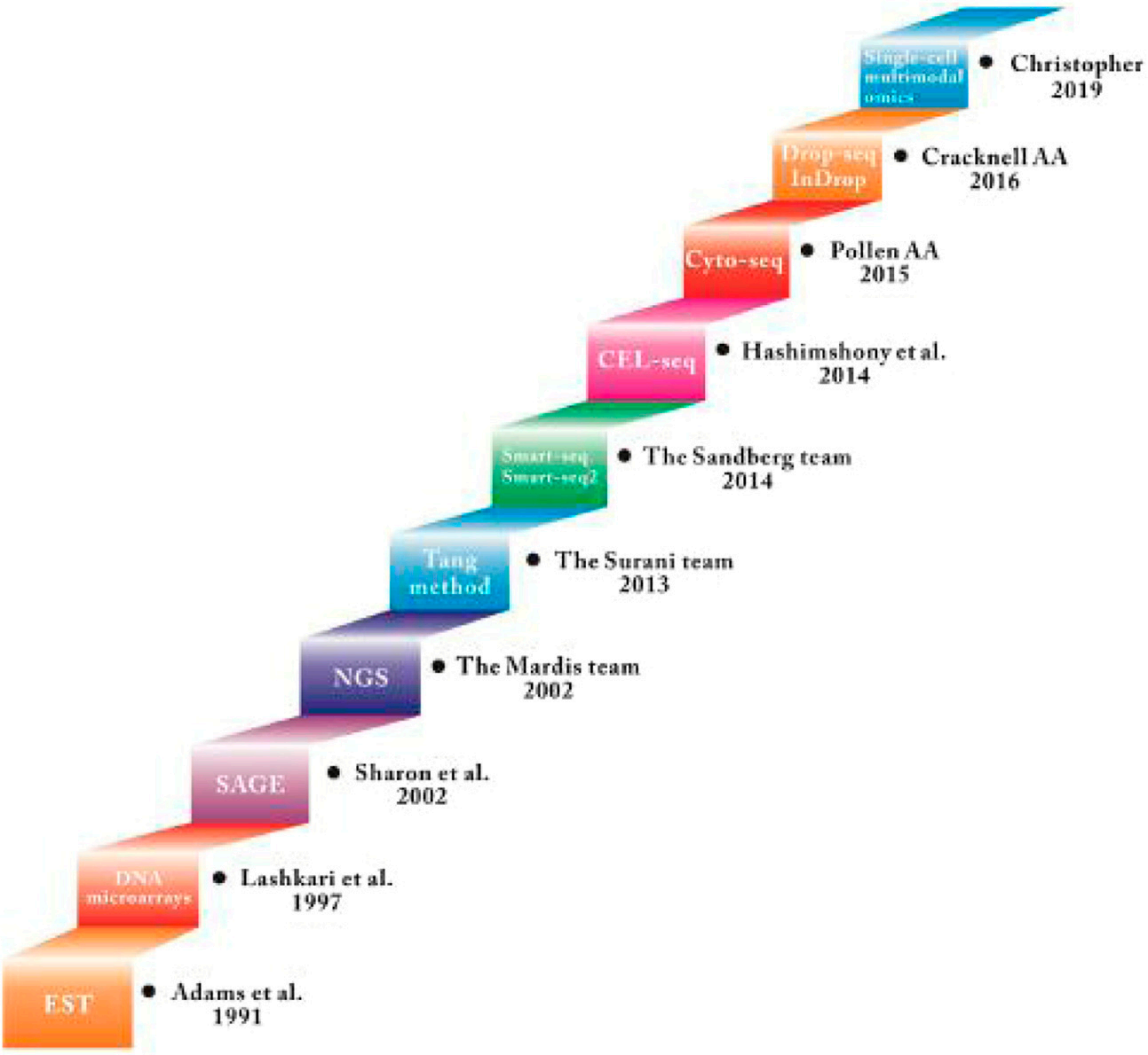
Summary of the development of Single-cell RNA sequecing technology.

In the field of ophthalmology, single-cell RNA sequencing research has been mostly applied to retina, from cell subtypes to targeted treatments for related diseases. Both humans and monkeys have fovea and macula, but mice are nocturnal dichromats and humans are diurnal trichromats. Therefore, studies on subtypes of retinal cells in humans and primates should ideally be published separately (Pauly et al., 2019). This review summarizes and discusses the latest progress and applications of scRNA-seq technology in the field of retina. So far, scRNA-Seq has been used in mouse, primate, human embryo and adult retinal tissue cell subtype research, as well as the pathogenic gene pathway research of various retinal-related diseases. In this review, we systematically reviewed the rapid progress of single-cell technology (Figure 1) and summarized the current challenges and unanswered questions in the field of retinal development and disease.

**GRAPHICAL ABSTRACT:**
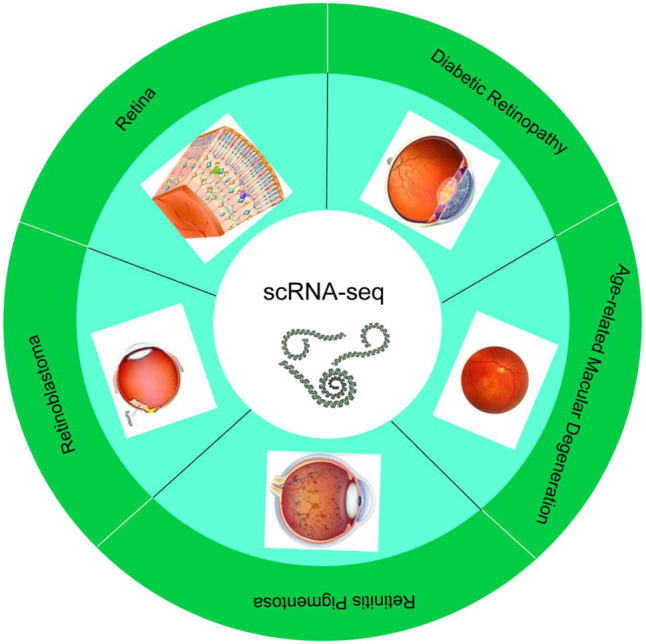


## Development of Single-Cell RNA Sequencing Technology

Single-cell transcriptome sequencing technology (scRNA-seq) is to analyze the expression profile of the cell transcriptome from the single cell level to identify cell-specific markers, discover rare cell types, cell subtypes, and reveal differences between cells expression (Zerti et al., 2020). The basic technical principles of scRNA-seq technology include: 1) separation technology, such as micromanipulation, laser capture microdissection, fluorescence activated cell sorting, 2) single-cell transcriptome amplification and sequencing library construction. Cells are the basic structural and functional units of organisms (Zerti et al., 2020). During their growth and development, due to different cell states and environmental stimuli, changes in transcriptome information show diversified manifestations. scRNA-seq can study the differential expression of RNA from a single cell level. Since the Tang team first applied scRNA-seq technology in 2009, scRNA-seq technology has received more research and development (Table 1). Besides, single-cell sequencing technology has been used to study stem cell differentiation, embryonic organ development, tumor tissue, immune tissue, nervous tissue, and other fields in recent years (Picelli, 2017). In the field of ophthalmology, it is mainly used to study the gene expression of normal retinal tissues and common retinal diseases, such as age-related macular degeneration and diabetic retinopathy.

**TABLE 1:** Principal characteristics of the most widely used scRNA-seq methods.

Name	Transcript coverage	Year	First discovery	Positional bias	Strand specificity	References
Tang method	Nearly full length	2013	Surani et al.	Strongly 3′	No	Gao, (2018)
STRT-seq	5′ only	2013	Sten linnarssons et al.	5′ only	No	Islam et al. (2012)
Smart-seq	Full length	2014	Sandberg et al.	Medium 3′	No	Valdes-Mora et al. (2018)
Smart-seq2	Full length	2014	Sandberg et al.	Weakly 3′	No	Valdes-Mora et al. (2018)
CEL-seq	3′ only	2014	Hashimshony et al.	3′ only	Yes	Hashimshony et al. (2012)
CEL-seq2	3′ only	2014	Hashimshony et al.	3′ only	Yes	Hashimshony et al. (2012)
MARS-seq	3′ only	2014	Hashimshony et al.	3′ only	Yes	Jaitin et al. (2014)
CytoSeq	Predefined genes only	2015	Pollen AA et al.	3′ only	Yes	Islam et al. (2014)
Drop-seq/InDrop	3′ only	2016	Cracknell JA et al.	3′ only	Yes	Pollen et al. (2014)
DroNC-seq	3′ only	2017	Habib et al.	3′ only	Yes	Habib et al. (2017)
Sci-RNA-seq	3′ only	2017	Cao et al.	3′ only	Yes	Cao et al. (2017)
Seq-well	3′ only	2017	Gierahn et al.	3′ only	Yes	Gierahn et al. (2017)
SPLiT-seq	3′ only	2018	Rosenberg et al.	3′ only	Yes	Rosenberg et al. (2018)
Quartz-Seq2	3′ only	2018	Sasagawa et al.	3′ only	Yes	Sasagawa Y et al. (2018)
Single-cell multimodal omics	Full length	2019	Christopher et al.	3′ only	Yes	Author Anonymous (2020); Moncada et al., 2020)

## Application of scRNA-Seq in Normal Retinal Tissue

In the field of ophthalmology, the single-cell RNA sequencing research in the past 5 years has mainly focused on the retina, and most of the research focuses on the exploration of cell subtypes, related genes and pathway. In particular, many researchers choose amacrine cells, bipolar cells and microglia cells for study. Among them, amacrine cells are the most diverse neurons, and most of them lack obvious molecular markers (Grunert and Martin, 1991), which has stimulated curiosity of various researchers in recent years. The retina is a highly heterogeneous tissue, and it is estimated that there are more than 100 nerve cell subtypes.

Primates (including humans) have a fovea on the retina, which is a small central area responsible for high vision and most color vision. However, the retina of mice does not have a fovea. This difference also limits some experimental studies. The distribution and number of primate and mouse retinal cells have a certain difference between the fovea and the periphery. Yi-Rong Peng et al. (Peng et al., 2019) used 165,000 single-cell RNA sequence maps to perform a comprehensive cell classification of the central fovea and peripheral retina of rhesus monkeys, of which 64 fovea (3 PRs, 2 HC, 12 BC, 27 AC, 16 RGC and four non-neurons) and 71 peripherals (2 PR, 2 HC, 11 BC, 34 AC, 18 RGC and four non-neurons) clusters. Comparison with the mouse retina type shows that the middle neuron type is tightly conserved, but the type and procedure of the projection neuron are different.

Based on the previous study, Wen-jun Yan et al. (Yan et al., 2020) compared the gene expression characteristics of human and cynomolgus monkey and fascicular monkey cell types. Besides, they identified five types of neurons (9,070 photoreceptors, 2,868 horizontal cells, 25,908 bipolar cells, 13,607 amacrine cells and 11,404 RGCs) and four types of non-neuronal cells. By comparing the retinal cell types of human and rhesus monkeys, the differentially expressed genes are summarized: the genes that are highly expressed in rhesus monkey retinal tissues are EPHX2, DB1 and DB6. GPATCH1 and CRHBP genes are highly expressed in human retinal tissues. In addition, by comparing retinal foveal cells with surrounding cells, they found that: 1) EPB41L2 and VTN are expressed by the fovea instead of the peripheral cone. 2) expression level of TTR in the fovea is higher than that of the surrounding bipolar types of DB3b and DB4. 3)TULP1 is expressed by peripheral but not foveal bipolar FMB and DB2. Besides, the transcriptome of approximately 85,000 cells from the fovea and surrounding retinas of seven adult donors was analyzed by Wen-jun Yan et al. (Yan W et al., 2020) using single-cell RNA sequencing (sRNA-seq) in 2020. The results showed that FOXP2+, FOXP1-, FOXP2+, FOXP1+, and F-RGCs were highly expressed in RGCS cell clusters. In addition, the comparison showed that more than 90% of the human types were transcriptionally consistent with those previously identified in macaques, and that the expression of disease-related genes was highly conserved between the two species. These results confirm the usefulness of macaques in simulating blinding diseases and provide a basis for investigating the molecular mechanisms of visual processing.

Regarding the study of fovea and peripheral cells, Sharon et al. (Banin et al., 2015) have reported 20 highly expressed genes in the macular region (such as SLC17A6, SNCG, NEFL, NET1, STMN2, YWHAH, UCHL1, DPYSL2, APP, NDRG4, TUBA1B,MDH1, EEF2) and 23 highly expressed genes in the peripheral region of the retina (SAG, RCVRN, UNC119, GPX3, PDE6G, ROM1, ABCA4, DDC, PDE6B, GNB1, NRL); based on bulk RNA Seq, Li et al. (Li et al., 2004) reported 1,239 The macular area is highly expressed and the 812 peripheral areas are highly expressed. These related studies provide a basic framework for single-cell analysis of species and across tissue regions.

In addition, scRNA-seq can be used to further study genetic markers and typing of specific optic nerve tissues and retinal cells. Macosko et al. (Macosko et al., 2015) analyzed the transcripts of 44,808 mouse retinal cells and identified 39 different transcribed cell populations, establishing a molecular map of gene expression for known retinal cell types and new candidate cell subtypes. Among them, 21 clusters of amacrine cells were mainly studied. 12 were identified as GABAergic (Gad1 and/or Gad2 positive), and the other five were glycine (Glcine transporter Slc6a9 positive). Ebf3 is a transcription factor found in SEG-glycine and nGnG-amacrine proteins and is specific for clusters 17 and 20.

To further study the gene expression of bipolar cells, Shekhar et al. (Shekhar et al., 2016) used mouse retinal bipolar cells (BCs) as the research object through DROP-SEQ and classified them by two different criteria. Firstly, according to whether the RBC is marked or not, a rod-shaped or cone-shaped BC is divided; secondly, according to the bipolar mark Isl1 and/or Grm6, the cone BC cluster can be further divided into on (3–6, 13, 15) and off (7–10, 12, 14) BC type. It is also worth mentioning that on the basis of the predecessors, the team further divided four types of BC5 (BC5A-BC5D), specifically BC5A (Sox6+) and BC5B (Chrm2+), BC5C (Slitrk5+), BC5D (Lrrtm1+).

On the basis of previous studies, O'Koren team (O'Koren et al., 2019) used single-cell sequencing to reveal the unique transcriptome-related genes of microglia in photoreceptor degeneration, such as Lsp1, asApoe, Ppiaf4, and Alox5ap, which were temporarily induced in the middle of the trajectory; Fabp5, Lgals3, Cd63, Lpl, Cybb, Mmp12, and Spp1 are adjusted up late in the trajectory.

The developmental pathways of mouse neural retina (NR) and retinal pigment epithelium (RPE) have been extensively revealed. However, the molecular mechanism of human NR and RPE formation and the interaction between these two tissues have not been well elucidated (Dulken et al., 2017). In recent years, some studies have used scRNA-seq technology to conduct experimental design with retinal multifunctional stem cells (RPCs) as the research object. RPCs are located in the inner layer of the optic cup (Oppikofer et al., 2017). They produced six types of neurons in retinal cells. The processes that retinal development needs to go through: RPC proliferation, cell fate determination, and specific neuronal differentiation (Gordon et al., 2013).

Yuqiong Hu et al. (Trimarchi et al., 2009) identified the main cell types of human fetal retina, which are RGC expressing γ-synuclein (SNCG), NEFL, ATOH7 and EBF3; HCs express ONECUT1/2/3; ACs express MEIS2, GAD1 and GAD2; BCs express VSX1 and VSX2; PCs express PDC, PDE6G, SAG, CRX and NRL; microglia express CX3CR1, C1QA, C1QB and C1QC; fibroblasts express COL3A1 and COL1A1.

According to the report, Xiying Mao et al. (Mao et al., 2019) found that the RPC specific markers VSX2 and PAX6 were co-expressed 28 days ago; after 28 days, the expression of VSX2 began to disappear on the central basal side of the retina, expressing the retinal ganglion cell (RGC) marker ELAVL3/The cells of four began to appear simultaneously, and the number of ELAVL3/4 positive cells gradually increased thereafter. HES1 and HES5 are briefly activated in RPC (Lukaszewicz and Anderson, 2011) and then suppressed in terminally differentiated neurons, and HES6 continues to be up-regulated after the lineage bifurcation point.

Based on the previous evidence, Brian S. Clark et al. (Clark et al., 2019) used single-cell RNA sequencing to describe ten developmental stages covering the entire process of retinal neurogenesis, our results indicate that NFI transcription factors (NFIA, NFIB, and NFIX) are selectively expressed in late RPCs and indicate that they regulate the fate of bipolar interneurons and Miller glial cells and promote proliferation and imactivation. Besides, Mariona Esquerdo-Barragán team (Esquerdo-Barragán et al., 2019) found that TOPORS, KLHL7, PRPF8, USP45, and Usp-20 were expressed at low levels in the retina through scRNA-seq. Josd1, Pan2, Usp11, Usp14, Usp15, Usp10, Usp22, Usp39 and cone cells Compared to the expression of rod differentiation, the expression of three genes (Otud7b, Usp46, and Usp48) increased in late cone cells; the expression of Usp45, Usp53, and Usp54 was limited to the photosensitive layer; Usp28, Usp37, or Otub1 is highly expressed in the embryonic period, but expression is stopped after birth; Usp12, Zranb1 or Usp32, its expression is extremely low in the embryonic period, but significantly increased before and after birth (Hojo et al., 2000). These genes are related to the ubiquitin proteasome system (UPS), which has important research significance for retinal precursor cell differentiation.

In order to further explain the tissue structure and cell subtypes of the chicken retina, based on previous studies, this year Masahito Yamagata et al. (Yamagata M and Sanes, 2021)used single-cell RNA sequencing (sRNA-seq) to generate a cellular atlas of chicken retinas (40,000 single-cell transcriptome), 136 cell types plus 14 sites or developmental intermediates were identified. The team mapped genes expressed in the majority of three types of retinal cells, namely VSX2 (CHX10) in the basal cells, TFAP2A in the central retinal cells, and RBPMS2 in retinal ganglion cells. The results provide new insights into the structure and evolution of the retina and lay the foundation for the study of the anatomy, physiology and development of the retina in birds.

For the past few years, the continuous application and development of scRNA-seq technology has been improved. The study of normal retinal cells in animals and human eyes can redefine the cluster of cells based on the marker gene (Table 2). It also enables a deeper understanding of tissue cells and subsequently the cluster of cells. Carrying out a more in-depth classification of cells helps to understand the heterogeneity of cells well, and also brings along a new perspective for our subsequent diagnosis and treatment of disease.

**TABLE 2:** New discoveries of genes and cell subtypes related to retina.

Study name	Methodology	Sample source	Number of cells sequenced	Year of publication	Molecules/pathways identified	References
Evan Z macosko et al.	Drop-seq	Mouse retinal cells	44,808	2015	Found 39 different cell populations	Macosko et al. (2015)
Shekhar et al.	Drop-seq	Mouse retinal bipolar cells	4 clusters	2016	Divided four types of BC5 (BC5A-BC5D)	Shekhar et al. (2016)
Yi-rong peng et al.	scRNA-seq	Macaque fovea and peripheral retina	165,000	2019	Fovea and peripheral retina contain more than 65 cell types	Peng et al. (2019)
Emily G O'Koren et al.	scRNA-seq	Mouse retina	4 clusters	2019	Found two types of microglia	O'Koren et al. (2019)
Sharon et al.	scRNA-seq	Mouse retina	6 clusters	2019	Reported reported 20 highly expressed genes in the macular region and 23 highly expressed genes in the peripheral region	Banin et al. (2015)
Li et al.	RNA-seq	10 non-proliferative DR patients and 11 non-DR T2DM patients	2051	2019	Found 1,239 areas the macular area is highly expressed and the 812 peripheral areas	Li et al. (2004)
Xiying mao et al.	scRNA-seq	Human embryonic stem cell (hESC)-derived 3D retinal organoids	16,348	2019	Found the RPC specific markers VSX2 and PAX6	Mao et al. (2019)
Yuqiong hu et al.	scRNA-seq	Human fetal NR and RPE	13,000	2019	Identified the main cell types of human fetal retina	Trimarchi et al. (2009)
The mariona esquerdo-barragán team	ChiP-seq	Mouse retina	87	2019	Found TOPORS, KLHL7, PRPF8, USP45, and Usp-20 were expressed at low levels in the retina	Esquerdo-Barragán et al. (2019)
Wen-Junyan et al. (2020)	scRNA-seq	Adult human donors	62,857	2020	Identified 5 types of neurons and 4 types of non-neuronal cells	Yan et al. (2020)
Wen-Junyan et al. (2020)	scRNA-seq	Adult human donors	85,000	2020	FOXP2+, FOXP1-, FOXP2+, FOXP1+, and F-RGCs were highly expressed in RGCS cell clusters	Yan W et al. (2020)
Masahito yamagata et al.	scRNA-seq	Chicken retinas	4,000	2021	VSX2 (CHX10) in the basal cells, TFAP2A in the central retinal cells, and RBPMS2 in retinal ganglion cells	Yamagata M and Sanes, (2021)

## Application of scRNA-Seq in Retinal Diseases

### scRNA-Seq in the Research of Targeted Therapy of Ocular Tumors Application

Different cells change differently at seperate stages of the disease. The transcriptome of many cell subtypes in the retina, especially rare cells, is usually obscured by a large number of RNA sequences. Therefore, understanding the transcriptome at the cell type or single cell level will expand research related to disease.

Single-cell RNA-seq can be used for targeted therapy of eye tumors (Kawaguchi et al., 2008). It is well established that the molecular and cellular characteristics of tumors can indicate the origin of tumor cells and provide a basis for targeted therapy. Retinoblastoma is a malignant tumor in infants and young children. In recent years, researchers have used single-cell sequencing technology to study the pathogenetic gene pathway and treatment of it.

Mcevoy et al. (McEvoy et al., 2011) performed single-cell gene expression array analysis on tumor cells of retinoblastoma patients and mouse models, showing that multiple cell types are specifically expressed in a single retinoblastoma cell. The results showed that human retinoblastoma expressed high levels of MDMX gene and MDMX protein. Some monoamine/catecholamine receptors in mice include serotonin receptors (HTR3A, HTR1E), dopamine receptors (DRD5) and histamine receptors (HRH3) Expression levels in retinoblastoma It is equal to or higher than the normal human retina.

Based on the previous study, Joseph Collin et al. (Collin et al., 2021) used nine human embryonic and fetal retinal tissues by sRNA-seq and ATAC sequence method. The results showed that Glu137Ter and Tyr655Ter were highly expressed in 4 month old embryonic tumor tissues. However, the Rb1c.763C and Arg255TER genes were overexpressed in embryonic tumor tissue at 34 months. In addition, CCNE1, CCNE2, CCNB2, CCNA2, and CDK1 genes were highly expressed in fetal tumor tissues. In addition, this study provides evidence of the heterogeneity of RB tumors and defines molecular pathways and new targeted therapeutic strategies.

### scRNA-Seq on the Pathogenesis of Age-Related Macular Degeneration and Treatment Research

In addition, single-cell sequencing technology also aids the study and treatment of retinal degenerative and vision loss diseases by analyzing the pathogenesis of related diseases and discovering new biological targets and markers.

Radeke et al. (Newman et al., 2012) discovered new age-related macular degeneration (AMD) biomarkers and gene expression characteristics of AMD pathogenesis. These findings indicate that the cell-based inflammatory response in the RPE choroid is a core feature of AMD. All AMD phenotypes in the RPE choroid are associated with high expression of all or a subset of the following chemokines, namely CXCL1, CXCL2, CXCL9, CXCL10, CXCL11, CCL2 and CCL8. AMD expression in retinal pigment epithelium Related bases and chemokines are C10orf18, ARL9, CXCL10, FZD10, CTSL2, CXCL. AMD may be a single disease with a common immune response process. The genes that regulate these immune activities, as well as many other genes found, represent promising new targets for the treatment and diagnosis of AMD.

On the basis of the previous research, Madhvi Menon et al. (Menon M et al., 2019) retinal cells were isolated and sequenced from six postmortem human retinal macular and surrounding panretinal suspension using droplet based microfluidic (20,091 cells) and nanopore based Seq Well (3,248 cells) to investigate cell types associated with age-related macular degeneration. The results showed that CFI, TIMP3, VEGFA and COL4A3 genes were highly expressed in AMD retinal cells.

Besides, Jones et al. (Jones et al., 2016) used human brain-derived neural precursor cells (hNPCs) to treat retinal degenerative lesions. The results showed that the top five genes with the greatest changes included Mir671, Lcn2, Cd74, Gfap, and Cebpd; Lcn2, Cd74, Gfap, and Cebpd (Hughes et al., 2003). All show that as retinal degeneration increases, Mir671, Lcn2, Cd74, and Cebpd play a role in the immune response of macrophages and/or microglia, suggesting that the activity of macrophages/microglia increases as the retina degenerates (Lawson et al., 1990).

### scRNA-Seq on the Pathogenesis of Diabetic Retinopathy and Treatment Research

In recent years, a series of studies on Diabetic Retinopathy (DR) have suggested that vision loss in DR patients is no longer considered to be a simple microvascular complication, also known as neurodegenerative disease (Kamalden et al., 2017). Different retinal cells, trophic factors, neurotransmitters, and inflammatory factors play an important role in the pathogenesis of diabetic retinopathy (Kamalden et al., 2017). Moreover, there are not many studies on diabetic retinopathy by single cell histology (Pastukh et al., 2019).

Xian Zhang et al. (Zhang et al., 2019) found that overexpression of AK077216 in DR patients resulted in downregulation of miR-383, but overexpression of miR-383 had no significant effect on the expression of AK077216; overexpression of AK077216 inhibited apoptosis of ARPE-19 cells (Ru et al., 2014), miR Overexpression of -383 plays the opposite role and attenuates the overexpression of AK077216; therefore it is concluded that AK077216 is down-regulated in diabetic retinopathy, and inhibits ARPE-19 cell apoptosis by down-regulating miR-383. Zimeng Li et al. found that miR-4448, miR-338-3p, miR-190a-5p, miR-485-5p and miR-9-5p are highly expressed in the serum of DR patients (Shaker et al., 2019).

### Application of scRNA-Seq Studies in Other Retinal Diseases

The types of neurons in the central nervous system are significantly different in terms of resilience to injuries or other injuries (Della Santina et al., 2013). In recent years, some researchers have provided a systematic framework to analyze the specific types of injuries through single-cell sequencing technology. Sexual response, and demonstrate that differential gene expression can be used to reveal the molecular targets of intervention.

Nicholas Tran et al. (Tran et al., 2019) first used single-cell RNA-seq (scRNA-seq) to generate a comprehensive molecular map of the 46RGC type in the adult retina. By tracking their survival after ONC (Optic Nerve Crush), the transcription and morphological changes before degradation were described, and each type of selectively expressed genes was determined. Among them, Igf1 (7/7 resRGCs), Opn4 (5/7) and Spp1 (3/7)/OE-Ucn, Ucn protein, OE-Timp2, KO-Crhbp, and KOMmp9 all promoted significant overall regeneration of the optic nerve.

Another experimental study, using single-cell sequencing technology, determined whether multiple explosion exposures caused greater damage to RGC than single explosion exposures (Hong et al., 2015). The results show that Cd40, Mrpl34, Kmo, Lmcd1, BC030870, I830077J02Rik, and Ms4a14 (Kim et al., 2008) are related genes that mediate neuroprotection.

scRNA-seq has been used as a comprehensive and fair method to study cell types and gene expression patterns in the retina of spontaneous, chronic and progressive autoimmune uveitis. Jacob et al. (Heng et al., 2019) used Aire−/− mice to establish a model of autoimmune uveitis retinitis. Mouse models offer a unique opportunity to study the mechanisms of autoimmune uveiretinitis, which is an important cause of vision loss. The team characterized 64,196 isolated retinal cells from eight samples using a droplet based sRNA-seq platform (10×genomics). The results showed that experimental uveiretinitis is a T-cell-driven disease, and the highly expressed genes in the following types of cells were: Th1 cells (T-bet+, IFNG+, CXCR6+, CD4+, CD8a, KLRA1), CD8a + T cells (CD8a^+^, CD4^−^, KLRA1), T follicular helper cells (BCL6+, CXCR5+, CD4^+^, CD8a) and regulatory T cells (Foxp3+, CD4^+^, IL10+). In addition, TGFb2 is the main TGF-β family member expressed in Aire mouse retina, mainly in the inner layer (INL). In conclusion, this study supports a similar central role of Th1 cells in Aire/uveoretitis, which has important implications for clinical treatment.

Besides, Wen-junYan et al. (Yu-Wai-Man et al., 2010) used cell atlas to evaluate the retinal expression of 1756 disease-related genes. Studies have shown that among the genes associated with retinitis pigmentosa (RP), RPGR and TOPORS, SLC25A46, SLC7A14 and RP9 are highly expressed in RGC (Delettre et al., 2002). In addition, RGR and RLBP1 are highly expressed in Müller glial cells. CRX, RAX2, GNAT2, PDE6H genes are highly expressed in rods and cones. RHO, NRL and NR2E3, all show the enrichment of the fovea (Miller et al., 2019). Lebers congenital amaurosis (LCA) is a group of severe hereditary retinal dystrophy, which is characterized by nystagmus, delayed or missing pupil light reflection, and blindness. Experimental results show that CEP290, GUCY2D and CRB1 genes are highly expressed in RGC (Anguita et al., 2021). In studies related to congenital quiescent night blindness (CSNB), it was found that GNAT1 and SLC24A1 were highly expressed in rod cells, while GRM6 and TRPM1 were highly expressed in bipolar cells (Clemons et al., 2013).

It can be seen that sc RNA-seq research can provide differentiated gene expression of cells for retinal diseases, transcription factor prediction, and the network communication interaction of each cell in the process of disease progression, which can provide new targets for the diagnosis and treatment of disease prediction. Using this technology, we can discover new cell subtypes and identify genetic markers of individual retinal subtype cells to help study and locate targets related to specific visual functions, thereby gaining a deeper understanding of cell function and cell heterogeneity explore the establishment of genetic networks that maintain cell diversity (Table 3).

**TABLE 3:** Studies of gene expression in retina diseases.

Study name	Methodology	Sample source	Diseases	Number of cells sequenced	Year of publication	Molecules/pathways identified	References
Justina McEvoy et al.	Single-cell gene expression array analysis	Human and mouse retina	Retinoblastoma	120	2011	Showed that there are multiple cell type-specific expressions in a single retinoblastoma cell	McEvoy et al. (2011)
Melissa K jones et al.	scRNA-seq	Rat retina	AMD	11,215	2016	Used human brain-derived neural precursor cells to treat retinal degenerative lesions	Jones et al. (2016)
Jacob S heng et al.	scRNA-seq	Rat retina	Autoimmune uveitis retinitis	64,196	2019	Defined the main immune effector cell types	Heng et al. (2019)
Nicholas M. Tran et al.	scRNA-seq	Mouse retinal ganglion cells	Optic nerve crush	46	2019	Generate a comprehensive molecular map of the 46RGC type in the adult retina	Tran et al. (2019)
Radeke et al.	scRNA-seq	Mouse retina	AMD	118	2019	Discovered new age-related macular degeneration (AMD) biomarkers and gene expression characteristics of AMD pathogenesis	Newman et al. (2012)
Xian Zhang et al.	RNA-seq	60 diabetic retinopathy patients	DR	383	2019	Found that overexpression of AK077216 in DR patients resulted in downregulation of miR-383	Zhang et al. (2019)
Madhvi menon et al.	scRNA-seq	Human retina	AMD	23,339	2019	CFI, TIMP3, VEGFA and COL4A3 genes were highly expressed in AMD retinal cells	Menon M et al. (2019)
Wen-Junyan et al. (2020)	scRNA-seq	Human retina	Retinitis pigmentosa	1756	2020	Used cell atlas to evaluate the retinal expression of 1756 disease-related genes	Yan W. et al. (2020)
Joseph collin et al.	scRNA-seq	Human retina	Retinoblastoma	655	2021	CCNE1, CCNE2, CCNB2, CCNA2, and CDK1 genes were highly expressed in fetal tumor tissues	Collin et al. (2021)

## Conclusion

Single-cell sequencing has opened up a new field to study different cell subtypes and genetic markers, and reveal the development mechanism and therapeutic targets of retinal-related diseases, and established itself as a valuable and unique tool to further study retinal tissue at the cellular level.

Single-cell sequencing can be used to study the classification of cell types and subtypes in the retina at the transcriptome level, and can help solve the heterogeneity and molecular complexity of the retina. An ideal scRNA-seq method can be used to analyze all coding and efficient non-coding cell transcripts, and even reveal subtle changes in gene expression. The past decade has witnessed significant technological development ever since the first scRNA-seq protocol was published in 2009 (Baden et al., 2016). With the steady decline in sequencing costs and the introduction of methods to significantly increase production every year, the genome, transcriptome, epigenome, and proteome of millions of cells can be sequenced simultaneously in the near future (Benowitz et al., 2017).

The main remaining problem is the challenge of efficiently separating individual cells from biological samples and analyzing large amounts of sequencing data. The close combination of scRNA-seq and bioinformatics technology can provide a powerful detection method to reveal the gene regulatory networks during cell development and differentiation. At present, the application of scrNA-seq in ophthalmic research is still limited. With the continuous progress of the technology, it may be rapidly expanded to the research of ocular diseases in the next few years.
